# Acute tubular injury versus acute interstitial nephritis in the kidney precision medicine project

**DOI:** 10.1172/jci.insight.204127

**Published:** 2026-08-10

**Authors:** Jennifer A. Schaub, Rajasree Menon, Ricardo Melo Ferreira, Elizabeth Kiernan, Insa M. Schmidt, Christine P. Limonte, Soumya Yennapureddy, Ying-Hua Cheng, Leal Herlitz, Avi Z. Rosenberg, Joel M. Henderson, Kelly D. Smith, Jeffrey B. Hodgin, Edgar Otto, Gilbert W. Moeckel, Lloyd G. Cantley, Suman Setty, Ulysses G.J. Balis, Dawit Demeke, Agnes B. Fogo, Andrew S. Bomback, Vivette D. D’Agati, Isaac E. Stillman, Jose R. Torrealba, Allen R. Hendricks, Erika Bracamonte, Vanessa Moreno, Pavan Bhatraju, Amy K. Mottl, Frank C. Brosius, Bijin Thajudeen, Steven G. Coca, Paul M. Palevsky, Parmjeet S. Randhawa, Raghavan Murugan, Laura Barisoni, Charles E. Alpers, Steven Menez, F. Perry Wilson, Dennis G. Moledina, Michael T. Eadon, Matthias Kretzler, Jonathan Himmelfarb, Chirag R. Parikh, the Kidney Precision Medicine Project

**Affiliations:** 1 Department of Internal Medicine, Division of Nephrology, and; 2 Department of Computational Medicine and Bioinformatics, Division of Nephrology, University of Michigan, Ann Arbor, Michigan, USA.; 3 Division of Nephrology, Department of Medicine, Indiana University School of Medicine, Indianapolis, Indiana, USA.; 4 Department of Medicine, University of Washington, Seattle, Washington, USA.; 5 Department of Medicine, Boston University Chobanian & Avedisian School of Medicine, Boston Medical Center, Boston, Massachusetts, USA.; 6 Hamberg Center of Kidney Health, University Medical Center Hamburg-Eppendorf, Hamburg, Germany.; 7 Department of Anatomic Pathology, Cleveland Clinic, Cleveland, Ohio, USA.; 8 Department of Pathology, Johns Hopkins University, Baltimore, Maryland, USA.; 9 Department of Pathology, Beth Israel Deaconess Medical Center and Harvard Medical School, Boston, Massachusetts, USA.; 10 Department of Laboratory Medicine and Pathology, University of Washington, Seattle, Washington, USA.; 11 Department of Pathology, University of Michigan, Ann Arbor, Michigan, USA.; 12 Department of Pathology, Yale University, New Haven, Connecticut, USA.; 13 Section of Nephrology, Department of Medicine, Yale University School of Medicine, New Haven, Connecticut, USA.; 14 Department of Pathology, University of Illinois at Chicago, Chicago, Illinois, USA.; 15 Department of Pathology, Microbiology and Immunology, Vanderbilt University Medical Center, Nashville, Tennessee, USA.; 16 Division of Nephrology, Department of Internal Medicine, and; 17 Department of Pathology, Columbia University Irving Medical Center, New York, New York, USA.; 18 Department of Pathology, Icahn School of Medicine at Mount Sinai, New York, New York, USA.; 19 Department of Pathology, Baylor College of Medicine, Houston, Texas, USA.; 20 Department of Pathology, University of Texas Southwestern Medical Center, Dallas, Texas, USA.; 21 Department of Pathology and Laboratory Medicine, University of Arizona, Tucson, Arizona, USA.; 22 Department of Pathology and; 23 Division of Nephrology, University of North Carolina, Chapel Hill, North Carolina, USA.; 24 Department of Medicine, University of Arizona, Tucson, Arizona, USA.; 25 Division of Nephrology, Icahn School of Medicine at Mount Sinai, New York, New York, USA.; 26 Renal-Electrolyte Division, Department of Medicine, University of Pittsburgh School of Medicine, Pittsburgh, Pennsylvania, USA.; 27 Kidney Medicine Section, VA Pittsburgh Healthcare System, Pittsburgh, Pennsylvania, USA.; 28 Department of Pathology, Thomas E. Starzl Transplant Institute, and University of Pittsburgh School of Medicine, Pittsburgh, Pennsylvania, USA.; 29 Department of Critical Care Medicine, University of Pittsburgh School of Medicine, Pittsburgh, Pennsylvania, USA.; 30 Department of Pathology, Division of AI and Computational Pathology, Department of Medicine, Division of Nephrology, Duke University, Durham, North Carolina, USA.; 31 Division of Nephrology, Johns Hopkins School of Medicine, Baltimore, Maryland, USA.; 32 Clinical and Translational Research Accelerator, Department of Medicine, Yale University School of Medicine, New Haven, Connecticut, USA.; 33 The Kidney Precision Medicine Project is detailed in Supplemental Acknowledgments.

**Keywords:** Immunology, Nephrology, Chronic kidney disease

## Abstract

**BACKGROUND:**

Acute interstitial nephritis (AIN) is a common cause of acute kidney injury (AKI), but the diagnosis may be missed as kidney biopsies are rarely obtained when acute tubular injury (ATI) is suspected.

**METHODS:**

The Kidney Precision Medicine Project is a cohort study that obtains kidney biopsies from individuals with AKI, which undergo pathologic and molecular interrogation. We compared ATI and AIN cases among the first 60 AKI participants.

**RESULTS:**

On clinicopathologic adjudication, 30 patients (50%) had a primary adjudicated diagnosis of ATI, 13 (22%) patients had AIN, 9 (15%) had diabetic nephropathy, and 3 (5%) had other conditions. There were increased interstitial white blood cells and tubulitis (*P* < 0.05 for both) in AIN compared with ATI. Prior to biopsy, the treating clinician suspected ATI in 83% of the cases with adjudicated ATI, while the treating clinician suspected AIN in 54% of the cases with AIN. Tissue transcriptomic signatures showed enrichment of proinflammatory signaling and increased expression of *CXCL9*, a chemokine induced by IFN-γ, in myeloid cells of participants with AIN. *CXCL9* localized to inflammatory infiltration in spatial transcriptomic data.

**CONCLUSION:**

Adjudication of kidney biopsies revealed distinct pathologic and molecular profiles between ATI and AIN. Kidney biopsy should be considered more frequently in AKI, as AIN is clinically underrecognized.

**TRIAL REGISTRATION:**

ClinicalTrials.gov NCT04334707.

**FUNDING:**

National Institute of Diabetes and Digestive and Kidney Diseases grants U01DK133081, U01DK133091, U01DK133092, U01DK133093, U01DK133095, U01DK133097, U01DK114866, U01DK114908, U01DK133090, U01DK133113, U01DK133766, U01DK133768, U01DK114907, U01DK114920, U01DK114923, U01DK114933, U24DK114886, UH3DK114926, UH3DK114861, UH3DK114915, and UH3DK114937.

## Abstract 2

This work demonstrates that acute interstitial nephritis, a potentially common cause of acute kidney injury, is sometimes not suspected by physicians.

## Graphical abstract

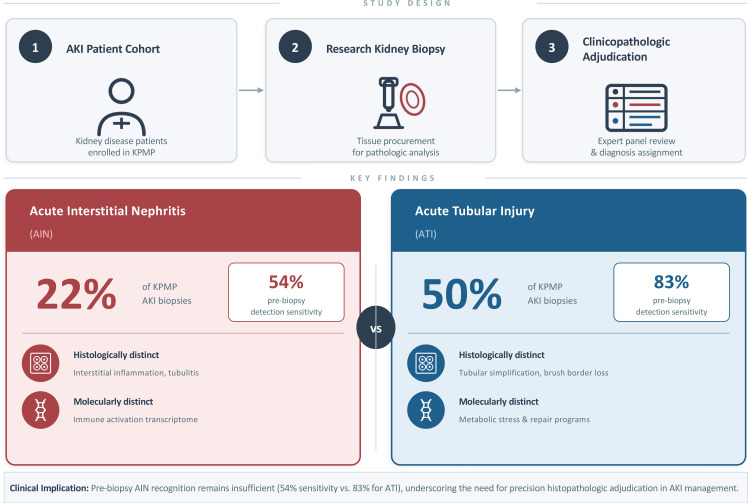

## Introduction

Acute kidney injury (AKI) affects approximately 10%–15% of hospitalized patients and up to 50% of those in intensive care units; it represents a major public health challenge with high morbidity and mortality (1–3). Among the diverse etiologies of AKI, acute tubular injury (ATI) and acute interstitial nephritis (AIN) are common histopathologic diagnoses (4). However, distinguishing between these entities is difficult without a kidney biopsy and has important therapeutic implications (5).

ATI, often resulting from kidney ischemia or nephrotoxin exposure, represents the most frequent cause of AKI in hospitalized patients. Its management is primarily supportive, focusing on addressing the underlying cause and providing renal replacement therapy when necessary. In contrast, AIN, frequently triggered by medications, infections, or systemic diseases, may benefit from specific interventions, including discontinuation of culprit medications and immunosuppressive therapy, particularly corticosteroids (6, 7). Early recognition and treatment of AIN is crucial for renal recovery and reducing chronic kidney disease (CKD) progression, as AIN-associated inflammation can rapidly progress to irreversible interstitial fibrosis and tubular atrophy (8).

Kidney biopsies can be used to distinguish between ATI and AIN but are rarely performed because of the risk of complications and the presumption that AKI is caused by ATI in the great majority of cases (9). This diagnostic uncertainty leads to incorrect management in many AIN cases and in some ATI cases. Previously proposed noninvasive diagnostic tools, such as urinary eosinophil testing, have demonstrated poor sensitivity and specificity (10). While recent studies have identified some promising urinary biomarkers for AIN diagnosis, none are yet available clinically to replace histologic evaluation (11, 12).

The Kidney Precision Medicine Project (KPMP) provides an unprecedented opportunity to address the diagnostic challenges of appropriate AKI diagnosis and treatment (13). By ethically obtaining kidney tissue from patients with AKI in the absence of traditional clinical indication for a biopsy, KPMP aims to improve disease classification, test noninvasive diagnostic biomarkers, and identify novel therapeutic targets (14, 15). In addition to traditional histopathology, KPMP uses spatial transcriptomics, single-cell RNA sequencing (scRNAseq) and other cutting-edge technologies to achieve these goals. All KPMP biopsies undergo a rigorous adjudication process to assign diagnoses that explain the underlying driver of the participant’s kidney disease.

We present here the first 60 participants with AKI in KPMP with completed adjudication data. We focused on comparing ATI and AIN to ascertain if the KPMP adjudication process produced categories that were meaningful at the histologic and molecular level and determine if kidney biopsies in a population that is sporadically biopsied provided clinically meaningful information.

## Results

### Participant characteristics.

There were 60 participants with completed clinicopathologic adjudication, with a mean age of 48 ± 15 years (Table 1). Of these, 72% were male and 47% were non-White participants. The baseline estimated glomerular filtration rate (eGFR) was 58 ± 5 mL/min/1.73 m^2^, and 28% of patients had an imputed baseline serum creatinine. Comorbidities included diabetes mellitus in 32% of participants and hypertension in 43% of participants. The average BMI was 29 ± 7 kg/m^2^. The majority of participants (66%) had Kidney Disease: Improving Global Outcomes (KDIGO) stage 3 AKI.

### Adjudicated diagnosis.

**Figure 1:**
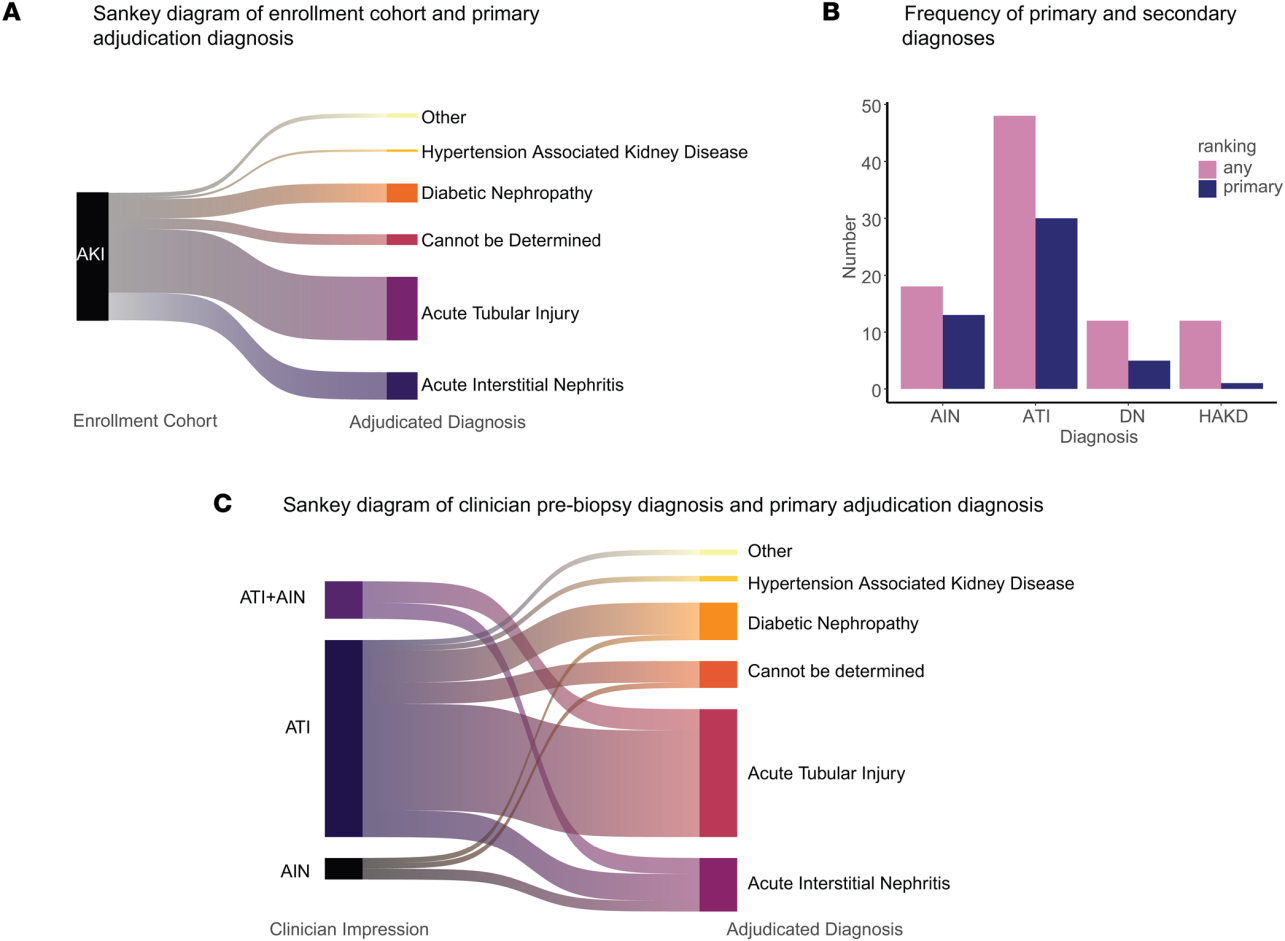
Adjudicated diagnosis in KPMP AKI participants. (**A**) Of participants enrolled with AKI, 50% had a primary diagnosis of ATI, 22% had AIN, 15% had DN, 2% had HAKD, and the remaining had other diagnosis or diagnoses that could not be determined. (**B**) Most participants had multiple disease processes in tissue, which are considered secondary diagnoses. Shown here are the percentage of participants with AIN, ATI, DN, or HAKD (primary or secondary). (**C**) Clinicians undersuspected the frequency of AIN. Of participants with adjudicated primary AIN and available prebiopsy impression data, 54% were suspected by the treating clinician prior to biopsy.

All kidney biopsies from KPMP were reviewed by two independent clinicians and two independent pathologists with access to the clinical presentation and pathology data. The case was then discussed by the adjudicating clinicians and pathologists, and a consensus was reached for primary and secondary adjudicated categories (Supplemental Figure 1; supplemental material available online with this article; https://doi.org/10.1172/jci.insight.204127DS1). The primary adjudicated diagnosis was deemed to be the disease that was the most likely underlying driver of the participant’s kidney disease. Upon completion of clinicopathologic adjudication, the primary disease process was determined to be ATI in 30 participants (50%), AIN in 13 participants (22%), diabetic nephropathy (DN) in 9 participants (15%), hypertension-associated kidney disease (HAKD) in 1 participant (2%), and other conditions in 2 participants (3%) (Figure 1A). These other conditions were granulomatous interstitial nephritis and IgA nephropathy. Consensus could not be reached on a primary adjudicated diagnosis for the remaining 5 participants, and there were no glomeruli in the biopsy cores for 1 of the participants. Almost all participants were found to have multiple diseases, which were considered “secondary” diagnosis, during the adjudication processes. When we combine primary and secondary diagnosis, we saw ATI in 48 (81%) participants, AIN in 18 (31%) participants, and DN in 12 (20%) of participants, and 12 (20%) participants had hypertensive/vascular-associated nephropathy present on biopsy (Figure 1B). For participants who had a primary adjudicated diagnosis of AIN, drug-induced injury was considered the most common etiology (7 of 13 or 58%).

### Prebiopsy clinician differential diagnoses undersuspect AIN.

Prior to the participant undergoing kidney biopsy, treating clinicians were asked to complete a survey indicating what histologic lesions they expected on the participant’s biopsy. Complete prebiopsy clinician impression data were available for 49 biopsies that had undergone adjudication. Of these 49 participants, 24 ultimately had a primary adjudicated diagnosis of ATN, 11 had a primary adjudicated diagnosis of AIN, 7 were diagnosed with DN, 5 could not be diagnosed, 1 was diagnosed with HACKD, and 1 had another diagnosis. Of patients with a primary adjudicated diagnosis of AIN, only 6 (54%) of them had AIN suspected by clinicians prior to biopsy (either as AIN alone or ATI+AIN), indicating that the sensitivity of a clinician to suspect AIN prior to biopsy was 54%. In comparison, of the 24 participants with a primary adjudicated diagnosis of ATI, ATI was suspected prior to biopsy by 20 of them, indicating that the sensitivity of a clinician to suspect ATI prior to biopsy was 83% (Figure 1C).

Correspondingly, of the 13 participants who had AIN suspected prior to biopsy (either as AIN alone or ATI+AIN), only 6 of them had AIN as the primary adjudicated diagnoses, suggesting a positive predictive value of 46%. Of the 38 participants who had ATI alone suspected prior to biopsy, 20 had ATI as the primary adjudicated diagnosis, indicating a negative predictive value of 53%.

### Histopathologic features distinguishing AIN from ATI.

**Figure 2:**
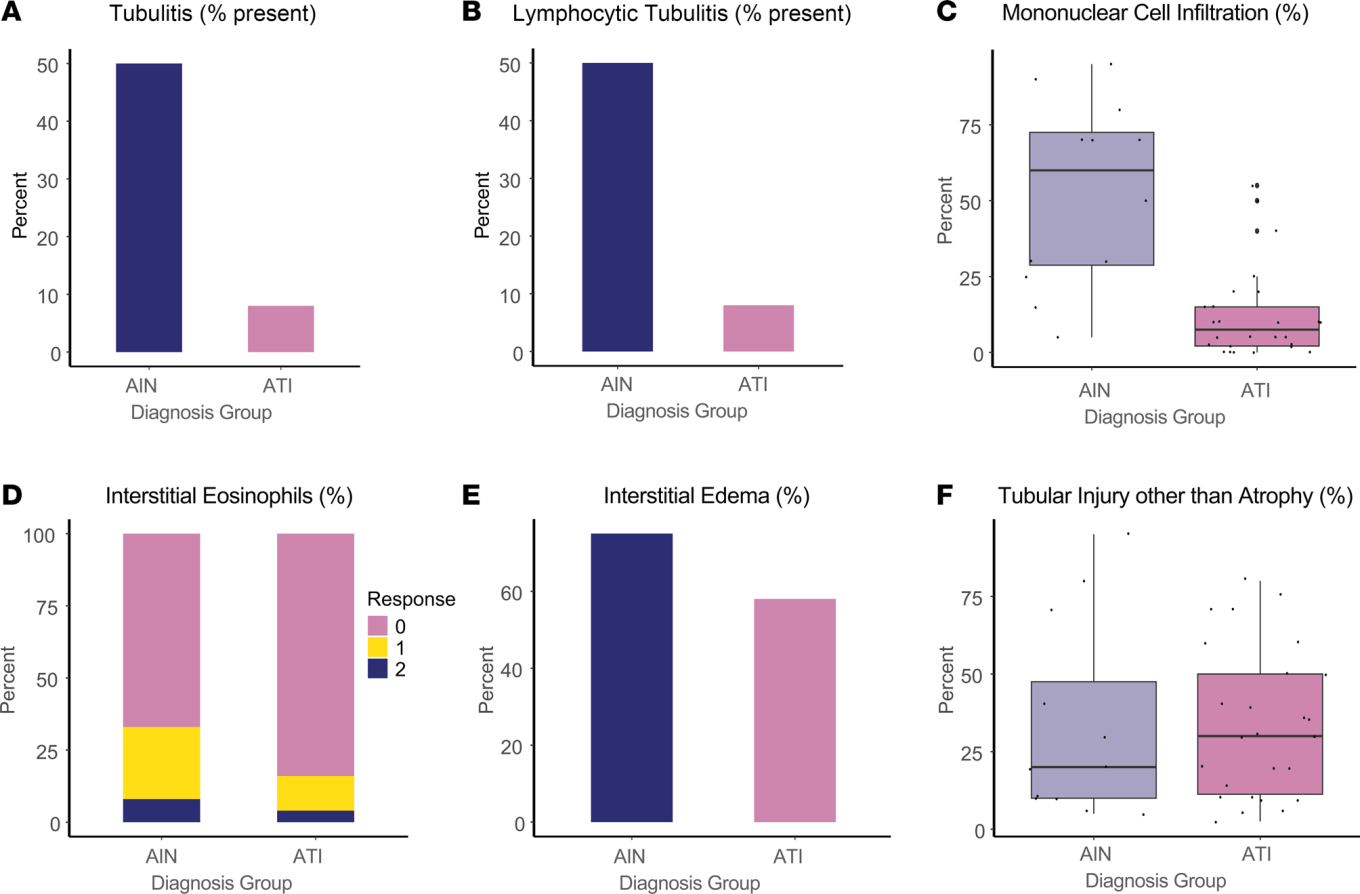
Descriptor features differ between the adjudicated categories of ATI and AIN. (**A**) Percentage of AIN and ATI cases with tubulitis. *P* < 0.01 (χ^2^ test). (**B**) Percentage of AIN and ATI cases with lymphocytic tubulitis. *P* < 0.001 (χ^2^ test). (**C**) Percentage of cortical interstitial mononuclear cell infiltrate in ATI and AIN cases. *P* < 0.05 (Wilcoxon’s rank sum). (**D**) Interstitial eosinophils. *P* was not significant. 0, no cortical involvement with interstitial eosinophils; 1, 1%–10% of cortex had interstitial eosinophils; 2, >10% had interstitial eosinophils (Fisher’s exact test). (**E**) Percentage of AIN and ATI cases with interstitial edema. *P* is not significant (χ^2^ test). (**F**) Percentage of cortical involvement of tubular injury other than atrophy in ATI and AIN cases. *P* is not significant (Wilcoxon’s rank sum).

As there are no consensus guidelines for histologic diagnosis of AIN (16), we sought to ascertain how histology in adjudicated primary AIN cases differed from adjudicated primary ATI cases. To do this, we compared histopathologic descriptor features from the two categories. Descriptor scoring is a more detailed and granular review of histologic features than the broader pathologic assessment and diagnosis performed in the adjudication process, and it occurs independently of the adjudication process. Descriptor features were scored in a subset of biopsies and were available for 26 biopsies with a primary adjudicated diagnosis of ATI and 12 biopsies with a primary adjudicated diagnosis of AIN. There was more tubulitis (Figure 2A) and lymphocytic tubulitis (Figure 2B) and a greater percentage of mononuclear cell infiltration (Figure 2C) in AIN cases compared with ATI cases (*P* < 0.05 for each). While there was a quantitatively greater amount of interstitial eosinophils (Figure 2D) and more interstitial edema (Figure 2E) in AIN cases, these differences were not statistically significant. Similarly, there was no significant difference in the amount of tubular injury other than atrophy (Figure 2F) between the ATI and AIN cases. Taken together, these results suggest that the adjudication process identified AIN cases that differed from ATI histologically.

### Molecular characterization using scRNAseq.

**Figure 3:**
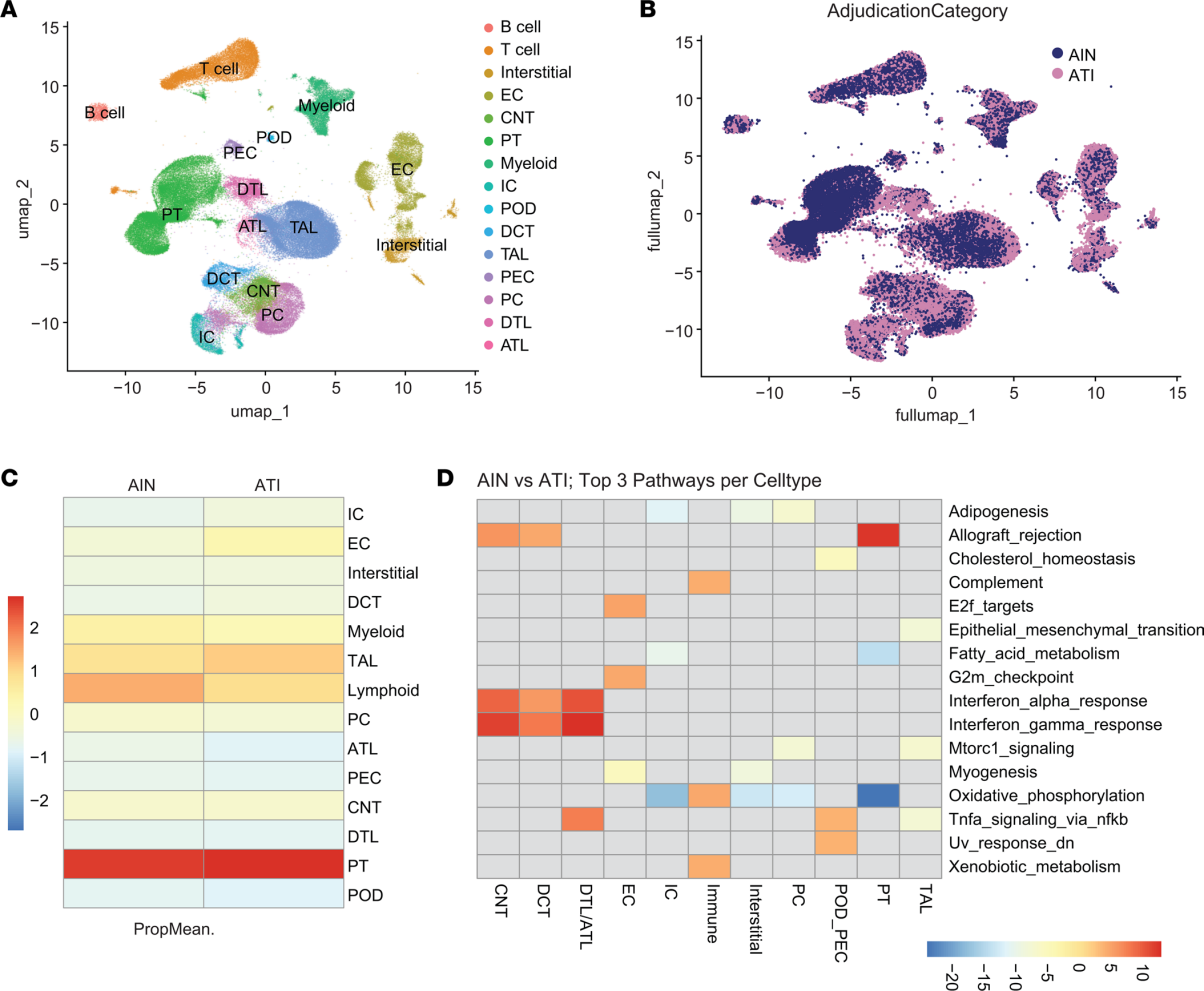
AIN cases have higher expression of signatures related to allograft rejection and *IFNG* signaling. (**A**) UMAP plot of samples (*n* = 10 for AIN, 57,014 cells; *n* = 10 for ATI, 42,336 cells). (**B**) ATI and AIN samples are well integrated. (**C**) Mean cell-type proportion in ATI+AIN and ATI-AIN samples. Lymphoid cells are enriched in ATI+AIN compared with ATI-AIN samples. (**D**) Top 3 MSigDB hallmark pathways enriched among the differentially expressed genes in major cell types from the ATI+AIN versus ATI-AIN analysis: *IFNG* response and allograft rejection were the most enriched pathways

scRNAseq data were available for 20 participants. Every participant with AIN also had coexisting histologic features of ATI on biopsy. Thus, we compared 10 samples with ATI without AIN (ATI-AIN, 42,336 cells) to 10 samples with ATI and AIN, where AIN may have been a primary or secondary adjudicated diagnosis (ATI+AIN, 57,014 cells) (Figure 3A). Each cell cluster had representation from the ATI-AIN and ATI+AIN cases (Figure 3B). The mean cell type proportion showed an enrichment of lymphoid cells in AIN compared with ATI (Figure 3C). In enrichment analysis using the Hallmark pathway database (17), the top differentially upregulated pathways in ATI+AIN included IFN-γ (*IFNG*) response and IFN-α (*IFNA*) response in distal/connecting tubules, complement activity in immune cells, and allograft rejection pathways in proximal tubular epithelial cells. Conversely, the top differentially downregulated pathway in ATI+AIN was oxidative phosphorylation in proximal tubular cells (Figure 3D).

For biomarkers that can be used for diagnosis of AIN, gene transcripts encoding *CXCL9* were most strongly expressed in myeloid cells of AIN cases, while *TNFA* and *IFNG* transcripts were most strongly expressed in T cells of AIN cases (Supplemental Figure 2).

### Spatial transcriptomic analysis.

**Figure 4:**
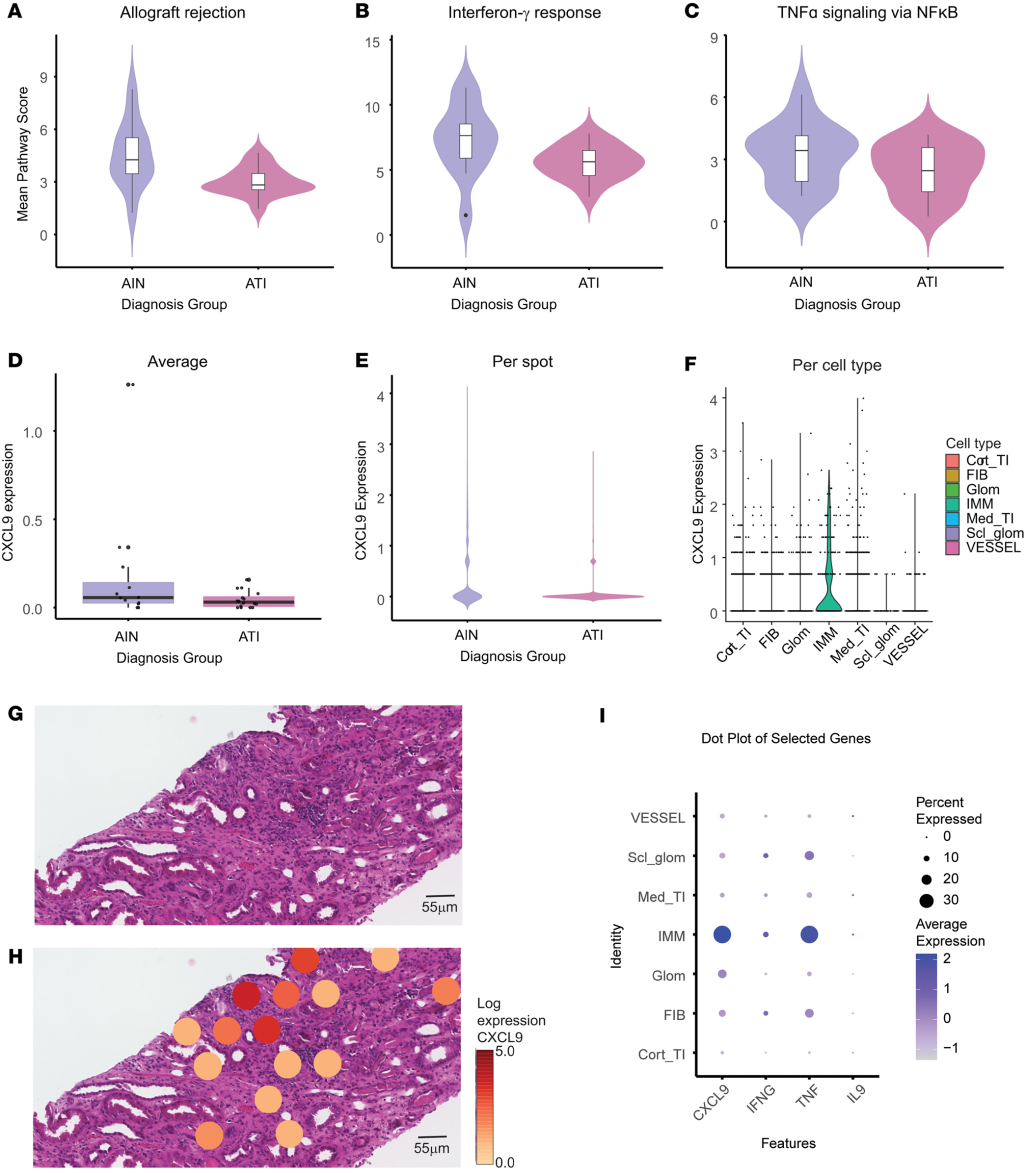
Spatial anchoring of immune-related pathways and genes. (**A**) Average pathway score of Hallmark allograft rejection in KPMP participants with AIN or ATI. (**B**) Average pathway score of Hallmark IFN-**γ** response of AIN or ATI. (**C**) Average pathway score of Hallmark *TNFA* signaling via *NFKB* of AIN or ATI. (**D**) Average expression of *CXCL9* per sample. (**E**) Expression of *CXCL9* per spot. (**F**) Expression level of *CXCL9* across histologically annotated injury regions: healthy cortical tubulointerstitium (Cort_TI), medullary tubulointerstitium (Med_TI), cortical tubulointerstitium with fibrosis (FIB) and cortical tubulointerstitium with immune cell infiltration (IMM), healthy glomeruli (Glom), and sclerotic glomeruli (Scl_glom). (**G**) Histological image demonstrating immune infiltration and injured tubules. Scale bar: 55 �m. (**H**) *CXCL9* expression mapped over histology of **G**. Scale bar: 55 �m. (**I**) Identification of marker genes across histological annotations.

We additionally confirmed our findings at the tissue level using two nonoverlapping spatial transcriptomic datasets from the KPMP (*N* = 28 frozen specimens with AKI) (15) and the Biopsy Biobank Cohort of Indiana (BBCI, *N* = 12 FFPE specimens) (18). We mapped pathway vectors for 3 immune-related pathways to all tubulointerstitial Visium spots of KPMP participants with adjudicated diagnoses of AIN (*N* = 12) or ATI (*N* = 16). Individuals with AIN had significant enrichment and upregulation of the allograft rejection pathway (*P* = 0.018, Figure 4A). *IFNG* response and *TNFA* signaling pathways trended in the same direction of effect (Figure 4, B and C). Due to its utility as a serum and urine biomarker of AIN, we queried the tissue expression of *CXCL9* on a per-patient and per-spot level (Figure 4, D and E). *CXCL9* expression was increased in the spots of individuals with AIN (*P* = 2.2 × 10^–16^) compared with those with ATI, with the same direction of effect in aggregated patient-level data.

To demonstrate a link between the molecular signatures and the histopathology, we next interrogated a complementary FFPE dataset derived from BBCI nephrectomies (*N* = 8) or specimens with CKD (*N* = 4) (19). While none of the 12 specimens carried a primary diagnosis of AKI, each had foci of tubular injury and immune infiltration. We histologically annotated every Visium spot according to 6 categories: healthy cortical tubulointerstitium, medullary tubulointerstitium, cortical tubulointerstitium with fibrosis, cortical tubulointerstitium with immune cell infiltration, healthy glomeruli, and sclerotic glomeruli. *CXCL9* expression was upregulated specifically in regions of immune cell infiltration (Figure 4, F–H). *TNFA* expression was similarly upregulated in these regions; both *CXCL9* and *TNFA* were more specific than *IL9* or *IFNG* expression (Figure 4I).

## Discussion

In KPMP, kidney biopsies of patients presenting with AKI demonstrated that AIN is underrecognized clinically. In clinical practice, these missed diagnoses could adversely impact patient care, as AIN is a potentially treatable condition. The adjudication process in KPMP reliably distinguished AIN and ATI, as evidenced by expected changes in histopathologic findings and the kidney transcriptome. Moreover, our analysis demonstrated that these missed cases of AIN reflect previously established pathophysiology and histologic features and reflect previously investigated biomarker profiles, suggesting that these cases would also benefit from commonly accepted management of AIN, including high-dose steroids and withdrawal of offending agents.

In KPMP, the most common primary adjudicated diagnosis for AKI participants was ATI followed by AIN, with frequent coexistence of both processes. This overlap underscores the complexity of AKI pathophysiology and highlights that many patients have multiple cooccurring pathologic disease processes contributing to their kidney disease. The rigorous KPMP adjudication process confirmed meaningful histopathologic distinctions, with primary AIN cases demonstrating significantly increased tubulitis and mononuclear cell infiltration compared with ATI cases. However, about 17% had a primary adjudicated diagnosis of either DN or HAKD. There are several possibilities regarding why ATI or AIN was not considered a primary diagnosis in patients presenting with AKI. First, these participants may have experienced prerenal azotemia without apparent tubular injury or tubular injury may have been missed due to sampling error. Additionally, most KPMP biopsies are obtained while the serum creatinine is downtrending, so it is possible that tubular injury features would have been apparent if the biopsy had been obtained earlier.

At the molecular level, our single-cell transcriptomic analysis revealed distinct transcriptomic signatures that align with the expected pathophysiology of ATI and AIN. The enrichment of *IFNG* signaling and allograft rejection pathways in AIN cases provides molecular validation of the immune-mediated nature of this disease (20–22). These pathways, traditionally associated with T cell–mediated immunity and transplant rejection, highlight the shared immunologic mechanisms underlying AIN regardless of etiology (20–22). The lymphoid cell enrichment indicates that the molecular signature of AIN is driven by immune cell infiltration rather than intrinsic tubular cell changes (21, 22).

Perhaps most important is our finding that AIN was underpredicted by treating clinicians prior to biopsy, which is consistent with reports from others (23). This systematic underrecognition has critical therapeutic implications, as delayed diagnosis of AIN is associated with incomplete recovery of kidney function and increased risk of CKD progression (13). While ATI management remains primarily supportive, AIN may benefit from specific interventions, including prompt discontinuation of culprit medications and consideration of immunosuppressive therapy (7, 8).

The molecular signatures we identified, particularly the upregulation of *CXCL9* in myeloid cells, suggest potential noninvasive diagnostic approaches. *CXCL9*, a chemokine induced by *IFNG*, has previously been identified as a promising urinary biomarker for AIN (11). Our spatial transcriptomic data demonstrating *CXCL9* expression patterns that correlate with areas of immune cell infiltration provide mechanistic support for its use as a biomarker and suggest it reflects active disease processes attributable to AIN rather than nonspecific inflammation.

A major strength of our study is that KPMP is one of the few cohorts with research kidney biopsies from patients who might not otherwise undergo a kidney biopsy. In addition, there are detailed clinical, pathologic and molecular data for each participant. However, several limitations merit consideration. First, our sample size of 60 participants may limit its generalizability. The enrichment for academic medical centers in KPMP recruitment may introduce selection bias toward more complex cases. Moreover, the exclusion criteria for KPMP may also introduce selection bias, resulting in an increased prevalence of AIN in KPMP. Second, the cross-sectional nature of our analysis precludes assessment of temporal changes in molecular signatures or their relationship to clinical outcomes. Longitudinal follow-up of this cohort will be essential to determine whether the identified molecular signatures predict response to therapy or long-term kidney functional outcomes. Third, while we identified established molecular signatures, translation to clinical practice and identification of novel mechanisms will require larger patient numbers, validation in independent cohorts, and development of practical, rigorously validated diagnostic assays.

Our findings highlight several priorities for future investigation. Prospective validation of urinary *CXCL9* and other identified biomarkers in larger, diverse cohorts is needed to establish diagnostic thresholds and performance characteristics. In addition, integration of these molecular findings with clinical risk factors could enable development of prediction models to identify patients most likely to benefit from kidney biopsies. In addition, our finding of frequent coexistence of AIN and ATI suggests that combined therapeutic approaches may be necessary, warranting clinical trials of immunosuppression in mixed phenotypes.

In conclusion, this work demonstrates that AIN and ATI represent distinct clinicopathologic entities with characteristic molecular signatures, yet AIN remains underrecognized in clinical practice. The enrichment of *IFNG* and allograft rejection pathways in AIN, driven predominantly by immune cell infiltration, provides both mechanistic insights and potential therapeutic and diagnostic targets. These findings underscore the value of kidney biopsy in selected patients with AKI and the urgent need for noninvasive diagnostics to enable timely recognition and treatment based on biological mechanisms of injury and repair. As the KPMP cohort expands and longitudinal outcome data become available, these molecular insights may ultimately transform our approach to AKI diagnosis and management.

## Methods

### Sex as a biological variable.

For this study, both sexes were included, but sex was not considered as a biological variable in the analysis.

### Overall design of KPMP.

The details of the KPMP study have been published previously (13). AKI participants are enrolled with a baseline eGFR ≥ 45 mL/min/1.73 m^2^, with a serum creatinine rise ≥1.5 times the baseline and urine microscopy consistent with ATI or an elevated kidney injury biomarker, were recruited from multiple sites across the United States. Imputed baseline eGFR was used for patients without a known baseline eGFR who had no known history of CKD. Exclusion criteria to ensure participant safety have been published previously (13). One kidney tissue core was used for standard histologic assessment by the recruiting site pathologist, and the remaining cores were used for molecular interrogation. Prior to reviewing biopsy results, the patient’s treating clinician provided prebiopsy diagnostic assessments of each participant’s AKI ascertained by independent interpretation of available clinical data. Clinicians are asked to select which histopathologic lesions they expect on a participant’s biopsy, and multiple selections are allowed. Race and ethnicity are self-reported by participants.

### Adjudication of biopsies.

To ensure accuracy and reproducibility of data, all kidney biopsies from KPMP were assigned an adjudicated clinicopathologic diagnosis (Supplemental Figure 1). The KPMP Biopsy Adjudication Committee is comprised of nephrologists and nephropathologists from across the consortium. The adjudication process consists of a structured clinical case presentation followed by histopathology review of light microscopy, immunofluorescence, and electron micrographs. Nephropathologists systematically grade glomerular, tubulointerstitial, and vascular compartment features. Two clinicians independently reviewed data regarding each participant’s clinical presentation and medical history, and two pathologists independently reviewed the histology images and electron micrographs obtained from the biopsy core and the recruiting site pathologist’s biopsy report, which also documented the results of routine immunofluorescence stains and ancillary immunohistochemistry studies when performed at the discretion of the recruitment site pathologist. The adjudicating clinicians and pathologists then discussed the case, and a consensus was reached for primary and secondary adjudicated categories. Following clinical and pathology data review and discussion, committee members select a consensus primary diagnosis from the following categories: “diabetic nephropathy,” “hypertension-associated kidney disease,” “acute interstitial nephritis,” “acute tubular injury,” “other,” or “cannot be determined”. DN was defined using Renal Pathology Society criteria (24). AIN was considered as interstitial inflammation, usually as an infiltrate of lymphocytes, monocytes/macrophages, and variable subpopulations of eosinophils, plasma cells, or neutrophils, often with concurrent features of interstitial edema. “Cannot be determined” is used for primary diagnosis in cases without predominant classical features of DN, HAKD, ATI, or AIN or clear evidence of alternative kidney pathology. The primary adjudicated diagnosis was deemed to be the disease that was the most likely underlying driver of the participant’s kidney disease. Adjudicators also provided information on the suspected etiology of AIN if applicable, with possible causes listed as “drug induced,” “autoimmune,” “other,” or “unknown”.

### Prebiopsy clinician impression.

Prior to kidney biopsy and independent of the adjudication process, the participant’s treating clinician was asked to complete a survey indicating what histologic lesions they expected on the participant’s kidney biopsy. Treating clinicians were allowed to select multiple options, and the options included the following: “acute tubular injury or acute tubular necrosis,” “acute interstitial nephritis,” “crystal nephropathy,” “cast nephropathy,” “acute glomerular disease,” “atheroembolic disease,” “thrombotic microangiopathy,” “acute vascular injury,” “hemodynamic or pre-renal AKI,” or “other.”

### Descriptor scoring.

The KPMP tubulointerstitial and vascular (TIV) descriptor scoring parameters were developed based on the NEPTUNE Digital Pathology Scoring System (25, 26). This form includes 64 unique TIV descriptors, of which 10 are scored as percentages observed across the cortex and medulla, while the remaining descriptors are assessed categorically within the cortex or medulla. Two KPMP pathologists — a primary scorer and a quality control (QC) scorer — review and score all stained sections (H&E, PAS, trichrome, and Jones silver histochemical stains, two of each) scanned into whole-slide images and stored in the KPMP Digital Pathology Repository, following the KPMP Manual of Operating Procedures (27). Scoring discrepancies between the primary and QC pathologists are discussed and resolved through adjudication, with a third pathologist consulted if necessary. These descriptors provide a comprehensive list of nonglomerular structural and cellular abnormalities observed in these regions. The definitions for these descriptors are publicly available on the KPMP website (https://www.kpmp.org/), and the subset of descriptors used for this analysis are shown in Supplemental Methods.

### scRNAseq and analysis.

scRNAseq and data processing were performed as previously described (14, 15). Sequence files were initially processed using Cell Ranger as part of the 10X Genomics pipeline. The resulting read count matrices were further corrected for ambient mRNA contamination with SoupX (v1.5.0). Downstream analysis was performed in Seurat (R package, version 5.0), with QC thresholds set at >500 and <5,000 genes per cell and <50% mitochondrial reads per cell. We have included QC plots (Supplemental Figure 3) relevant to mitochondrial reads per cell in the samples studied. For each sample group, normalization, variable gene identification, and principal component analysis were conducted independently. Sample integration was accomplished using reciprocal principal component analysis, followed by unsupervised clustering. Aggregate raw read counts at cell-type level were generated from the integrated single-cell dataset using the AggregateExpression function embedded in Seurat (v5) package. The differential gene expression analysis between ATI+AIN and ATI-AIN groups was performed using DESeq2 (28), where AIN was either a primary or secondary adjudicated diagnosis. Finally, enrichment analysis of pathways was conducted by deCoupleR R package using the MSigDB Hallmark database (17), with processes showing adjusted *P* < 0.05 considered significant.

### Spatial transcriptomics.

Pathway analysis was done across all the Visium Frozen samples with decoupleR (v.2.12.0) mlm function (29). Pathways Hallmark allograft rejection, Hallmark IFN-**γ**, and Hallmark *TNFA* signaling via *NFKB* of AIN versus ATI were extracted from msigdbr database (https://www.gsea-msigdb.org/gsea/msigdb/collections.jsp). Average and standard deviation pathway scores were evaluated per sample. The statistical significance (*P* value) of each pathway between AIN and ATI was obtained with a 2-tailed *t* test. *CXCL9* mRNA expression was extracted from the expression matrix across all the spots and then averaged across all the samples. The expression of the *CXCL9* gene was extracted across all histological annotations of the tissue samples. The images in Figure 4, G and H, with immune infiltration and injured tubules of a DN sample were extracted from the loupe browser(v.8.1.2) along with the display of *CXCL9* expression.

### Statistics.

Pathology descriptor features were compared using χ^2^ test, Fisher’s exact test, or Wilcoxon’s rank sum as appropriate. All statistical analyses were completed in SAS v9.2, and a *P* value of less than 0.05 was considered significant.

### Study approval.

Experiments on human samples followed all relevant guidelines and regulations. Human samples collected as part of the KPMP consortium (https://www.kpmp.org/) were obtained with written informed consent and approved under a protocol by the KPMP single IRB of the University of Washington Institutional Review Board (IRB#20190213). Samples from the BBCI (18) were acquired under waiver of consent as approved by the Indiana University Institutional Review Board (IRB # 1906572234). 

### Data availability.

All data from KPMP are publicly available at (https://www.kpmp.org/doi-collection/10-48698-2q2k-vz55). The following sample IDs were used in the single-cell analysis presented in Figure 3: AIN, 30-10018, 30-11080, 32-10074, 30-10929, 30-11104, and 936-10217 and ATI, 30-10034, 30-10125, 30-11141, 30-11154, 32-10003, 32-10419, 33-10005, 33-10006, 34-10187, 34-10478, and 933-10155. Data from the BBCI are available at Visium FFPE GEO GSE307817.

## Author contributions

Designed research studies: JAS, MTE, MK, JH, and CRP. Acquired data: RMF, EK, LH, AZR, JMH, KDS, JBH, EO, GWM, SS, UGJB, DD, ABF, ASB, VDD, IES, JRT, ARH, EB, VM, PB, AKM, FCB, BT, SGC, PMP, PSR, R Murugan, LB, CEA, SM, FPW, DGM, MTE, and CRP. Analyzed data: JAS, R Menon, RMF, EK, IMS, CPL, SY, YHC, and MTE. Wrote the manuscript: JAS, R Menon, EK, LGC, MTE, MK, JH, and CRP.

## Conflict of interest

R Murugan received research grants from National Institute of Diabetes and Digestive and Kidney Diseases (NIDDK) and Vantive Healthcare Inc. and consulting fees from Vantive Healthcare Inc., Baxter Inc., AM Pharma Inc., Bioporto Inc. La Jolla Inc., Novartis Inc., and Fresenius Medical Inc. MK reports grants and contracts through the University of Michigan from Chan Zuckerberg Initiative, AstraZeneca, NovoNordisk, Eli Lilly, Boehringer-Ingelheim, European Union Innovative Medicine Initiative, Certa Therapeutics, RenalytixAI, Regeneron, NovoNordisk, Sanofi, Dimerix, Travere and Vera Therapeutics. MK has received consulting fees through the University of Michigan from NovoNordisk, Alexion, Novartis, Roche Diagnostics, and Vera Therapeutics. In addition, MK has a patent (PCT/EP2014/073413 “Biomarkers and methods for progression prediction for chronic kidney disease”) licensed. LGC serves as a consultant for DropShot Therapeutics. DGM and CRP are named coinventors on a pending patent (“Methods and Systems for Diagnosis of Acute Interstitial Nephritis”) and cofounders of the diagnostics company Predict AIN LLC that has entered into a technology license and development agreement with Machaon Inc. DGM serves as a consultant for Biohaven Inc. JMH has received research support from Evotec, Novo-Nordisk, Pfizer, and Visterra and serves as a consultant with Novartis and has received fees from them. FPW reports funding from AHRQ R01HS027626; research support from Amgen and Boeringher-Ingelheim, and consultancy fees from WndrHlth. ABF serves on an advisory board for Vera. CRP is an inventor on a pending patent, “Reduce Discard of Kidneys for Transplantation after Brain Death using Repair Biomarkers,” that has been licensed to Eurofins Viracor. JH is a founder, CEO, and equity owner in Kuleana Technologies (Kuleana Technologies is focused on developing technologies to improve renal dialysis and other renal replacement therapies utilized for patients with end-stage renal disease.) and is a named inventor of IP (prohealing elastic angiogenic microporous vascular graft, system, and method for removing uremic toxins from a patient’s body, water-conserving hemodialysis system, zwitterionic copolymer coatings, and related methods) filed through the University of Washington and licensed to Kuleana Technologies. He serves on advisory boards for Maze Therapeutics, Aplagon, Vera Therapeutics, Barcode Nanotech, and Quris AI and has research collaborations with Intus Biosciences, AstraZeneca, and Aurinia Pharmaceuticals.

## Funding support

This work is the result of NIH funding, in whole or in part, and is subject to the NIH Public Access Policy. Through acceptance of this federal funding, the NIH has been given a right to make the work publicly available in PubMed Central.

- NIDDK grants U01DK133081, U01DK133091, U01DK133092, U01DK133093, U01DK133095, U01DK133097, U01DK114866, U01DK114908, U01DK133090, U01DK133113, U01DK133766, U01DK133768, U01DK114907, U01DK114920, U01DK114923, U01DK114933, U24DK114886, UH3DK114926, UH3DK114861, UH3DK114915, and UH3DK114937 to KPMP.

## Supplementary Material

Supplemental data

undefined

ICMJE disclosure forms

Supplemental data set 1

Supporting data values

## Figures and Tables

**Table 1:**
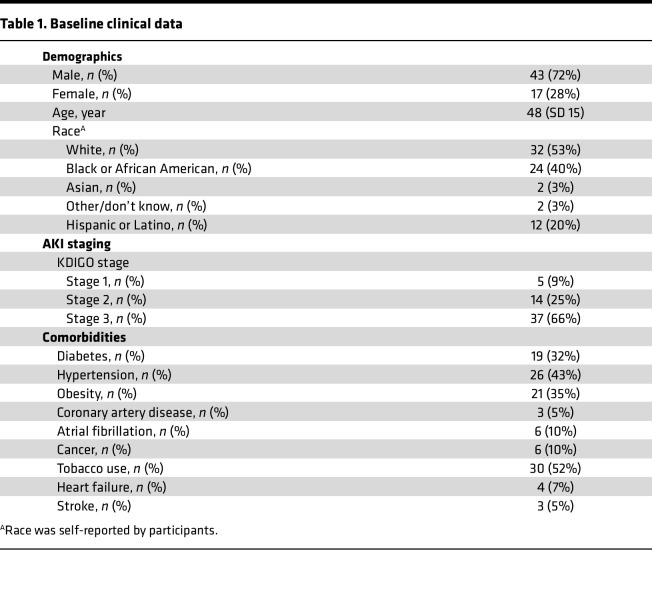
Baseline clinical data

## Version 1 08/10/2026

Electronic publication
