# Supplementary material for: Acute tubular injury versus acute interstitial nephritis in the kidney precision medicine project
Source: JCI Insight. 2026 Aug 10;11(15):e204127. doi: 10.1172/jci.insight.204127 (PMC13464007; doi:10.1172/jci.insight.204127)
Supplement: Supplementary file 1 [file jciinsight-11-204127-s312.pdf]

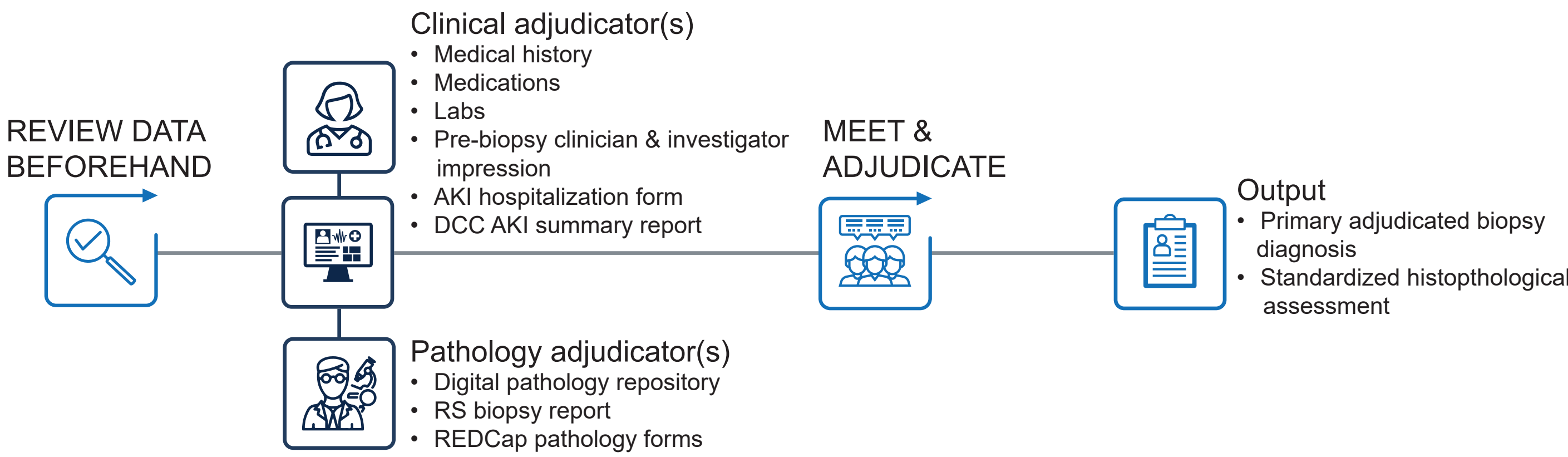

Supplementary Figure 1. Adjudication Workflow. Flow diagram demonstrating the KPMP adjudication process.

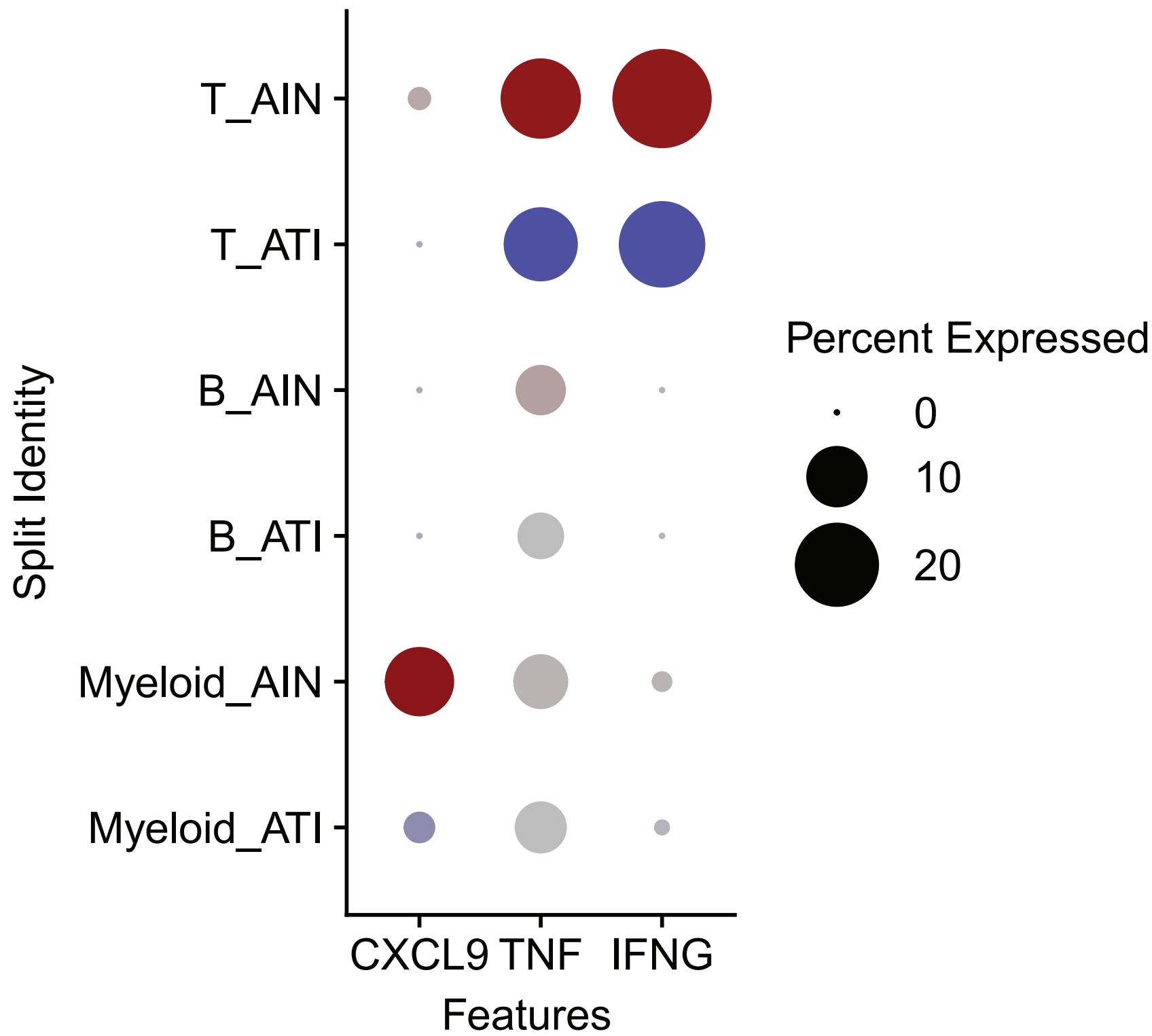

Supplementary Figure 2. Expression of AIN biomarkers. Dot plot showing expression of previously investigated biomarkers of AIN.

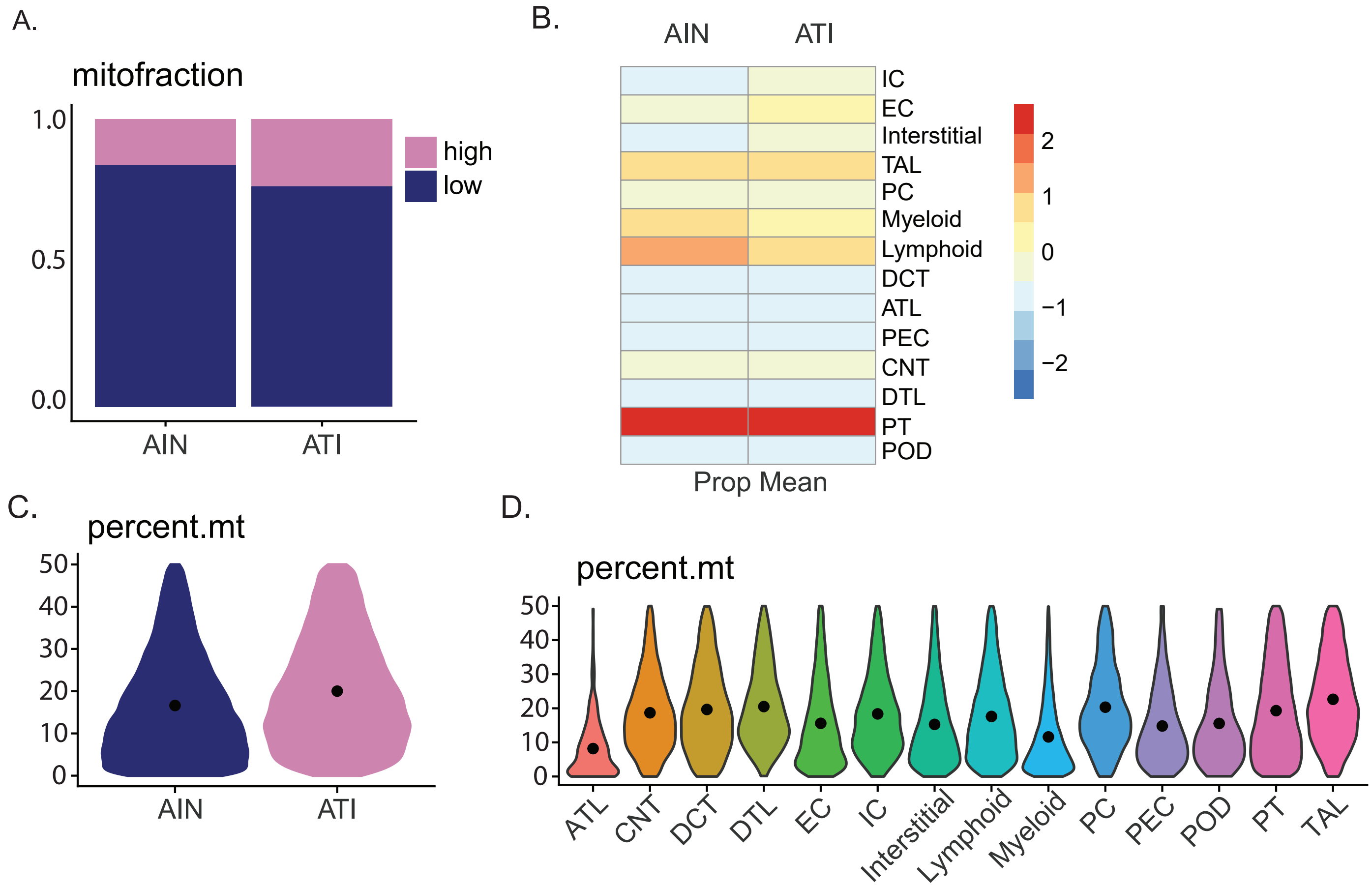

Supplementary Figure 3. **QC plots summarizing mitochondrial reads per cell in AIN and ATI groups. A.** Distribution of cells in AIN and ATI groups by mitochondrial read levels (>30% vs ≤30%). **B.** Mean cell type proportions in AIN and ATI (column-scaled) samples excluding the cells with >30% mitochondrial reads. Mean cell type proportions in AIN and ATI (column-scaled) samples, excluding cells with >30% mitochondrial reads, are similar to those observed when all cells were included (Figure 3C). This indicates that cells with high mitochondrial reads are correctly assigned to appropriate cell types rather than forming one distinct low-quality cluster. **C.** Violin plots comparing the distribution of mitochondrial reads per cell in AIN and ATI groups, with a slight increase observed in AIN, potentially reflective of disease biology. **D.** Violin plots illustrating the distribution of mitochondrial reads across identified cell types, with average mitochondrial reads per cell around 20% or less for all cell types.
